# Saliva Quantification of SARS-CoV-2 in Real-Time PCR From Asymptomatic or Mild COVID-19 Adults

**DOI:** 10.3389/fmicb.2021.786042

**Published:** 2022-01-03

**Authors:** Florence Carrouel, Emilie Gadea, Aurélie Esparcieux, Jérome Dimet, Marie Elodie Langlois, Hervé Perrier, Claude Dussart, Denis Bourgeois

**Affiliations:** ^1^Health, Systemic, Process, UR4129 Research Unit, University Claude Bernard Lyon 1, University of Lyon, Lyon, France; ^2^Equipe SNA-EPIS, EA4607, University Jean Monnet, Saint-Etienne, France; ^3^Clinical Research Unit, Emile Roux Hospital Center, Le Puy-en-Velay, France; ^4^Department of Internal Medicine and Infectious Diseases, Protestant Infirmary, Caluire-et-Cuire, France; ^5^Clinical Research Center, Intercommunal Hospital Center of Mont de Marsan et du Pays des Sources, Mont de Marsan, France; ^6^Department of Internal Medicine and Infectious Diseases, Saint Joseph Saint Luc Hospital, Lyon, France; ^7^Clinical Research Unit, Protestant Infirmary, Lyon, France

**Keywords:** SARS-CoV-2, COVID-19, saliva, viral load, virus isolation, real-time reverse transcription PCR, infectivity, quantitative

## Abstract

The fast spread of COVID-19 is related to the highly infectious nature of SARS-CoV-2. The disease is suggested to be transmitted through saliva droplets and nasal discharge. The saliva quantification of SARS-CoV-2 in real-time PCR from asymptomatic or mild COVID-19 adults has not been fully documented. This study analyzed the relationship between salivary viral load on demographics and clinical characteristics including symptoms, co-morbidities in 160 adults diagnosed as COVID-19 positive patients recruited between September and December 2020 in four French centers. Median initial viral load was 4.12 log_10_ copies/mL (IQR 2.95–5.16; range 0–10.19 log_10_ copies/mL). 68.6% of adults had no viral load detected. A median load reduction of 23% was observed between 0–2 days and 3–5 days, and of 11% between 3–5 days and 6–9 days for the delay from onset of symptoms to saliva sampling. No significant median difference between no-symptoms vs. symptoms patients was observed. Charge was consistently similar for the majority of the clinical symptoms excepted for headache with a median load value of 3.78 log_10_ copies/mL [1.95–4.58] (*P* < 0.003). SARS-CoV-2 RNA viral load was associated with headache and gastro-intestinal symptoms. The study found no statistically significant difference in viral loads between age groups, sex, or presence de co-morbidity. Our data suggest that oral cavity is an important site for SARS-CoV-2 infection and implicate saliva as a potential route of SARS-CoV-2 transmission.

## Introduction

The fast spread of COVID-19 is related to the highly infectious nature of SARS-CoV-2. The disease is suggested to be transmitted through saliva droplets and nasal discharge (Li Y. et al., 2020). Indeed, SARS-CoV-2 is found in nasopharyngeal secretions, and its viral load is consistently high in the saliva, mainly in the early stage of the disease (Sapkota et al., 2020). In addition to saliva secreted by the major or minor salivary glands, saliva samples also contain secretions from the nasopharynx or from the lungs through the action of cilia lining the airway (Huang et al., 2021). Saliva can have potential applications in the context of COVID-19 by direct detection of the virus, quantification of the specific immunoglobulins produced against it, and for the evaluation of the non-specific, innate immune response of the patient (Ceron et al., 2020). Up to date, several cross-section and clinical trial studies published support the potential of detecting SARS-CoV-2 RNA in saliva as a biomarker for COVID-19, providing a self-collection, non-invasive, safe, and comfortable procedure (Caixeta et al., 2021; Carrouel et al., 2021).

Most published RT-qPCR assays for the diagnosis of SARS-CoV-2 are qualitative (Han et al., 2021). Considering the variability of viral load between and within patients, quantification of absolute viral load directly from pure raw saliva is important for COVID-19 diagnosis and monitoring (Vasudevan et al., 2021). Quantification of viral load has other objectives compared to screening. It gives an indication of the degree of contagiousness, provides guidance on the interest to predict contagiousness of patients and hence to guide epidemiological decisions, to evaluate different samples from different anatomical locations, to predict the patient prognosis and assess disease progression (Jacot et al., 2020). In this context, evidence reports on the salivary viral load or duration of viral detection or infectivity of COVID-19 to trace the disease and to implement strategies aimed at breaking the chain of disease transmission are particularly important (Huang et al., 2021). Overall, the studies were of low-to-moderate quality given that most of the included studies comprised case series and case reports at the early stages of the pandemic (Nasserie et al., 2021). Furthermore, given the number of studies analyzing the Chinese population, it is quite possible that the results cannot be generalized to other populations, especially to asymptomatic cases linked to 80% of disease transmission (Walsh et al., 2020). There is consensus that these results should be viewed with caution and should be confirmed by larger, more robust studies. It is clear that more research is needed on this topic to correlate viral load with symptoms and clinical outcomes (Mahallawi et al., 2021).

Hart et al. (2021) showed that about 65% of virus transmission occurs before symptoms develop. Thus, individuals circulate in the general community before their infections are detected (Hart et al., 2021). To better understand SARS-CoV-2 infectiousness before symptoms develop or with mild symptoms, we analyzed salivary viral load at the moment of disease. The purpose of this study is to outline the salivary SARS-CoV-2 viral load over the course of the infection in asymptomatic or mild COVID-19 adults.

## Materials and Methods

### Study Design and Subjects

This analysis was a part of the randomized controlled trial BBCovid protocol published (Carrouel et al., 2020) and registered at ClinicalTrials.gov (NCT04352959). One hundred and sixty subjects diagnosed as COVID-19 positive patient were recruited between September and December 2020 in four French hospital centers. The study protocol was reviewed and approved by the National Ethics Committee “Committee for the Protection of Persons South Mediterranean III,” France (2020.04.11 sept _20.04.06.46640). Written informed consent was obtained from all enrolled individuals. The study was conducted in accordance with the Declaration of Helsinki and the International Conference on Harmonization–Good Clinical Practice guidelines.

The inclusion criteria were: (i) age 18–85 years-old, (ii) RT-PCR positive COVID-19 nasopharyngeal swab, (iii) asymptomatic or mild clinical symptoms for less than 8 days.

The exclusion criteria were: (i) pregnancy, (ii) breastfeeding, (iii) risk of infectious endocarditis, (iii) use of mouthwash regularly (more than once a week), (iv) inability to comply with protocol, (v) lack of written agreement, (vi) unable to answer questions, and (vii) uncooperative patients.

### Collecting of Demographic Data and Clinical Characteristics of Subjects

During the inclusion visit, demographic data (age, sex) and clinical characteristics (co-morbidities, symptoms, date of apparition of symptoms…) were collected from the subjects. Reporting of the nature of the symptoms was recorded at the time of the viral load measurement. An electronic medical record (e-CRF) permitted to record all these informations.

### Quantification of SARS-CoV-2 Salivary Load

#### Saliva Sampling

At 9 a.m., each subject collected one salivary sample with an accredited health care professional using the Saliva Collection System kit (Greiner Bio-one, Kremsmünster, Austria). Only one sample was collected from each adult. Firstly, with the saliva extraction solution, the subject rinsed the oral cavity for 2 min. Secondly, the subject spit into a saliva-collection beaker, without coughing or scraping to clear the throat, to collect the non-stimulated and pure saliva (between 1 and 3.5 mL). Finally, the saliva sample transferred to a sterile tube and stored at 4°C until analysis.

#### Quantification of SARS-CoV-2 Viral Load by Real-Time RT-PCR

RNA was extracted from 200 μL of saliva sample using the NucliSens easyMAG instrument (bioMérieux, Marcy-l’Etoile, France), according to the manufacturer’s guidelines. The RNA was eluted in 50 μL of water and used as a template for real-time (rt) RT-PCR.

Real-time RT-PCR assays were performed with the Invitrogen Superscript™ III Platinum One-Step qRT-PCR system (Invitrogen, Illkirch, France). The mix was composed of 5 μL of extracted RNA, 1 μL of Superscript III RT/Platinum Taq Mix, 12.5 μL of 2X reaction buffer, 0.4 μL of a 50 mM magnesium sulfate solution, 1 μL of RdRp-IP2 forward primer (0.4 μM), 1 μL of RdRp-IP2 reverse primer (0.4 μM), 1 μL of RdRp-IP4 forward primer (0.4 μM), 1 μL of RdRp-IP4 reverse primer (0.4 μM). The primer and probe sequences correspond to the RdRp-IP2 and the RdRp-IP4 assays designed at The Institut Pasteur to target a section of the RdRp gene (nt 12621-12727 and 14010-14116 positions) based on the sequences of SARS-CoV-2 (NC_004718) made available on the Global Initiative on Sharing All Influenza Data data-base on 11 January 2020 (Table 1). These primers and probes were manufactured by Eurofins (Genomics, Germany).

**TABLE 1:** RT-PCR for the detection of SARS-CoV-2: primers and probes used.

Name	Sequences (5′–3′)	PCR product	References
***RdRp gene/nCoV_IP2***			
nCoV_IP2-12669Fw	ATGAGCTTAGTCCTGTTG	108 pb	CNR*
nCoV_IP2-12759Rv	CTCCCTTTGTTGTGTTGT		CNR*
nCoV_IP2-12669bProbe(+)	[5′]HEX-AGATGTCTTGTGCTGCCGGTA-[3′]BHQ-1		CNR*
***RdRp gene/nCoV_IP4***			
nCoV_IP4-14059Fw	GGTAACTGGTATGATTTCG	107 pb	CNR*
nCoV_IP4-14146Rv	CTGGTCAAGGTTAATATAGG		CNR*
nCoV_IP4-14084Probe(+)	[5′]Fam—TCATACAAACCACGCCAGG—[3′]BHQ-1		CNR*

*CNR: National Reference Center for Respiratory Viruses, Institut Pasteur, Paris, France.

The assays were performed on a Quant Studio 5 (Thermo Fisher Scientific, Dardilly, France) with the following program: 55°C for 10 min (reverse transcription), followed by 95°C for 2 min, and then 45 cycles of 95°C for 3 s and 58°C for 30 s. Each run included three negatives’ samples bracketing unknown samples during RNA extraction, two positive controls, and one negative amplification control. When a sample was positive for RdRp-IP4, the quantification of the number of RNA copies was performed according to a scale ranging from 10^2^ to 10^6^ copies per μL. The SARS-CoV-2 viral load in saliva was calculated as the number of RNA copies per mL of saliva.

### Statistical Analysis

SPSS Windows 20.0 (IBM, Chicago, IL, United States) was used for the descriptive statistics median values and interquartile range (IQR) and mean values with SD for the quantitative variables and percentages for categorical variables. One-way ANOVA was used to compare the differences of median between groups in a univariate analysis. Binary logistic regression analysis was used to model the relationship between viral load values as the dependent variable and the other parameters were entered individually as independent variables (adjusted for age and gender). The detailed statistical methods are indicated in the table footnotes. All data were considered statistically significant when *p* < 0.05.

## Results

### Demographic Data and Clinical Characteristics of Subjects

The sex, the age and the clinical assessments of the study group are summarized in Table 2. The sample consisted of 160 subjects (55.97% of females and 44.03% of males) with a mean age of 43.62 ± 15.56 years. One co-morbidity was declared by 22.15% of subjects. The median time between symptoms onset and the positive nasopharyngeal RT-PCR was 4 days [IQR 3–5]. The median number of symptoms per subjects was 4 [IQR 2–5]. The most frequent symptoms described by 52.87% of subjects were the cough and the headache followed by myalgia in 48.41% of subjects. Fever was reported in 41.40% of subjects; anosmia in 40.13% of subjects; ageusia in 38.22% of subjects; dyspnea in 12.74% of subjects and; gastro intestinal symptoms in 8.92% of subjects. The absence of symptoms was only observed in 8.92% of subjects. For symptomatic subjects, the saliva sample was collected in median 6 days [IQR 5–7] after the onset of symptoms.

**TABLE 2:** Baseline characteristics of enrolled patients with Coronavirus Disease 2019.

Variable	
Gender, n/N* (%)	
Male	70/159 (44.03%)
Female	89/159 (55.97%)
**Age (years)**	N = 158
Mean ± SD	43.62 ± 15.56
Median [IQR]	43 [30–55]
Age (class), n/N* (%)	
18–34 years	52/158 (32.91%)
35–54 years	65/158 (41.14%)
55–77 years	41/158 (25.95%)
Co-morbidity, n/N* (%)	33/149 (22.15%)
**Time between positive RT-PCR and symptom onset (days)**	N = 153
Mean ± SD	3.93 ± 1.46
Median [IQR]	4 [3–5]
Time between positive RT-PCR and symptom onset (class), n/N* (%)	
0–2 days	27/153 (17.65%)
3–5 days	108/153 (70.59%)
6–8 days	18/153 (11.76%)
**Number of symptoms per subjects**	N = 157
Mean ± SD	3.71 ± 2.27
Median [IQR]	4 [2–5]
Number of symptoms per subjects (class), n/N* (%)	
None	14/157 (8.92%)
1–4	40/157 (25.48%)
5–6	65/157 (41.40%)
7–9	38/157 (24.20%)
Symptoms, n/N* (%)	
None	14/157 (8.92%)
Fever	65/157 (41.40%)
Cough	83/157 (52.87%)
Dyspnea	20/157 (12.74%)
Headache	83/157 (52.87%)
Myalgia	76/157 (48.41%)
Gastro symptoms	14/157 (8.92%)
Anosmia	63/157 (40.13%)
Ageusia	60/157 (38.22%)
**Delay from symptom onset to saliva sampling (days)**	N = 141
Mean ± SD	5.55 ± 1.62
Median [IQR]	6 [5–7]
Delay from symptom onset to saliva sampling (class), n/N* (%)	
0–2 days	7/141 (4.96%)
3–5 days	57/141 (40.43%)
6–9 days	77/141 (54.61%)

*n, Number of subjects, N, Total number of subjects.*

*n/N, Number of subjects/Total number of subjects.

### Quantification of SARS-CoV-2 Viral Load According to Demographics and Clinical Characteristics of Participants

Median initial viral load was 4.12 log_10_ copies/mL (IQR 2.95–5.16 log_10_ copies/mL, range 0–10.19 log_10_ copies/mL). The first quartile (Q1) corresponded to a viral load starting at 2.95 log_10_ copies/mL, whereas the second (Q2) corresponded to a viral load starting at 4.12 log_10_ copies/mL, and the third (Q3) corresponded to a viral load starting at 5.16 log_10_ copies/mL.

**FIGURE 1:**
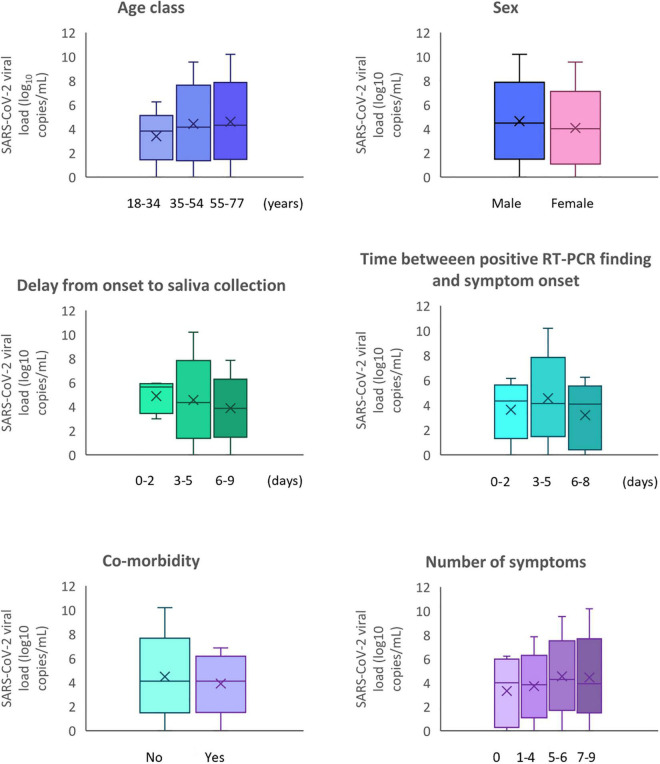
SARS-CoV-2 viral load according to demographics and clinical characteristics of participants.

**FIGURE 2:**
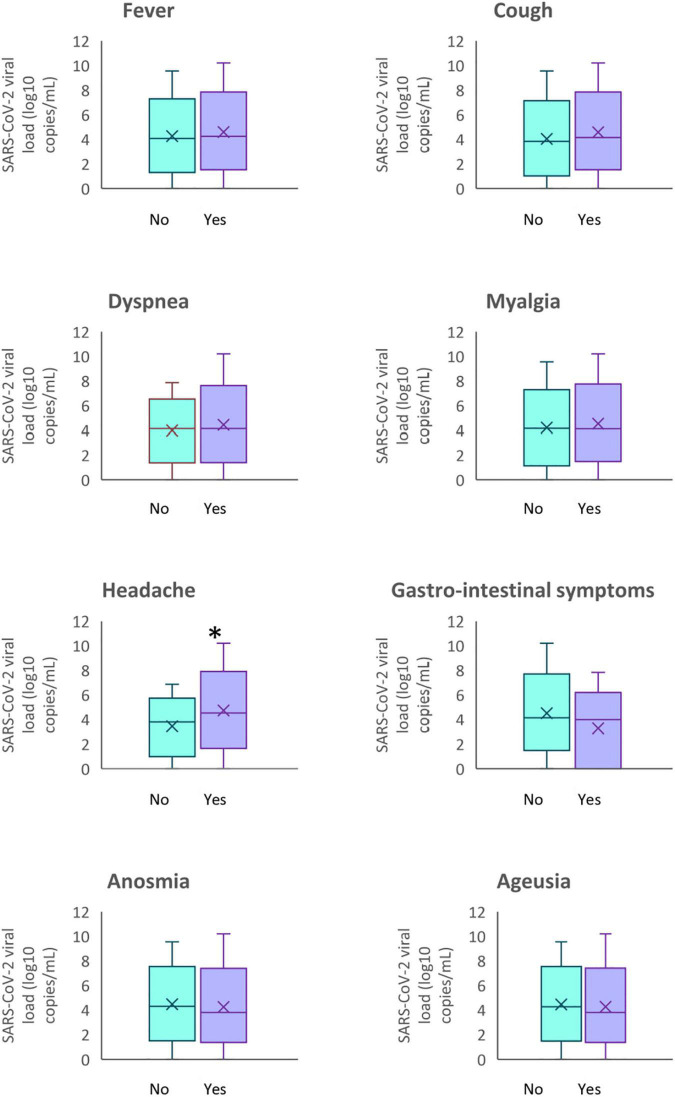
SARS-CoV-2 viral load according to the symptoms of participants. *p < 0.05.

The SARS-CoV-2 salivary viral load according to demographics and clinical characteristics of participants are described in Table 3 and Figures 1, 2. The median SARS-CoV-2 salivary viral load increased not significantly with age groups from 3.82 log_10_ copies/mL (18–34 years) to 4.31 log_10_ copies/mL (55–77 years). Salivary viral load was not associated with sex. The presence of co-morbidity or not, did not significantly modify the SARS-CoV-2 salivary viral load. Same observation was done for the time between RT-PCR and symptom onset, the delay from symptom onset to saliva sampling and for the number of symptoms per participants. Patients with symptoms demonstrated a median initial viral load of 4.12 log_10_ copies/mL (IQR 2.95–5.16; range 0–10.19 log_10_ copies/mL), while the no-symptoms patients had indices of 4.01 log_10_ copies/mL (IQR 0.56–5.75 log_10_ copies/mL, range 0–6.24 log_10_ copies/mL).

**TABLE 3:** Association of viral Load with demographics and clinical parameters for all enrolled participants: N: number of subjects.

Variable	N	Viral load, median [95% CI]	*p*-value
**Sex**			0.290^a^
Male	70	4.48 [2.99–5.54]	
Female	89	4.02 [2.18–4.69]	
**Age**			0.290^b^
18–34 years	52	3.82 [2.89–4.57]	
35–54 years	65	4.14 [2.72–5.72]	
55–77 years	41	4.31 [2.94–5.62]	
**Co-morbidity**			0.856^a^
No	116	4.12 [2.95–5.15]	
Yes	33	4.12 [3.01–5.5]	
**Time between positive RT-PCR and symptom onset**			0.637^b^
0–2 days	27	4.33 [2.62–5.09]	
3–5 days	108	4.13 [2.94–5.48]	
6–8 days	18	4.08 [0.81–4.82]	
**Delay from symptom onset to saliva sampling**			0.206^c^
0–2 days	7	5.63 [3.91–5.88]	
3–5 days	57	4.34 [2.75–5.47]	
6–9 days	77	3.86 [2.96–4.72]	
**Number of symptoms per subjects**			0.573^b^
1–4	40	3.85 [2.16–4.75]	
5–6	65	4.29 [3.4–5.49]	
7–9	38	3.93 [2.98–5.16]	
None	14	4.01 [0.56–5.75]	
**Symptoms**			
Fever			0.504^a^
No	92	4.07 [2.6–5.01]	
Yes	65	4.24 [3.04–5.47]	
Cough			0.097^a^
No	74	3.83 [2.05–4.74]	
Yes	83	4.14 [3.03–5.48]	
Dyspnea			0.627^a^
No	137	4.12 [2.75–5.17]	
Yes	20	4.13 [2.77–5.04]	
Headache			0.004^a^
No	74	3.78 [1.95–4.58]	
Yes	83	4.51 [3.32–5.59]	
Myalgia			0.721^a^
No	81	4.14 [2.25–5.02]	
Yes	76	4.10 [2.98–5.29]	
Gastrointestinal symptoms			0.212^a^
No	143	4.12 [2.95–5.17]	
Yes	14	3.88 [0–4.57]	
Anosmia			0.126^a^
No	94	4.29 [2.95–5.5]	
Yes	63	3.78 [2.7–4.54]	
Ageusia			0.147^a^
No	97	4.26 [2.94–5.49]	
Yes	60	3.78 [2.71–4.59]	

*^a^Mann-Whitney test.*

*^b^Kruskal-Wallis test.*

*^c^ANOVA test.*

### Determination of Factors Associated With SARS-CoV-2 Salivary Viral Load

The analysis of the mean difference of SARS-CoV-2 salivary load was reported in Table 4. For the delay from onset of symptoms to saliva sampling, the SARS-CoV-2 salivary load continuously decreased with the of the days. A median load reduction of 23% was observed between 0–2 days and 3–5 days, and of 11% between 3–5 days and 6–9 days. However, no significant difference between the groups was detected.

**TABLE 4:** Association of viral load with sex, age, symptoms of severe acute respiratory syndrome coronavirus 2 (SARS-CoV-2) infection: univariable analysis.

Variable	References level	Class level	Mean difference [95% CI]	*p*-value
**Age**	CV		0.02 [−0.01–0.04]	0.179
**Age (class)**	18–34	35–54	0.40 [−0.40–1.20]	0.330
		55–77	0.42 [−0.48–1.32]	0.359
**Gender**	Male	Female	−0.60 [−1.28–0.08]	0.084
**Co-morbidity**	No	Yes	−0.01 [−0.83–0.81]	0.974
**Time between positive RT-PCR and symptom onset**	CV		−0.11 [−0.35–0.13]	0.354
**Time between positive RT-PCR and symptom onset (class)**	0–2 days	3–5 days	0.26 [−0.67–1.18]	0.583
		6–8 days	−0.39 [−1.70–0.92]	0.560
**Delay from symptom onset to saliva sampling**	CV		−0.12 [−0.34–0.10]	0.283
**Delay from symptom onset to saliva sampling (class)**	0–2 days	3–5 days	−0.87 [−2.55–0.81]	0.312
		6–9 days	−1.31 [−2.96–0.35]	0.124
**Number of symptoms per subjects**	CV		0.08 [−0.07–0.24]	0.275
**Number of symptoms per subjects (class)**	None	1–4	0.02 [−1.31–1.36]	0.973
		5–6	0.56 [−0.71–1.83]	0.387
		7–9	0.50 [−0.85–1.85]	0.468
**Symptoms**				
None	Yes	No	−0.39 [−1.60–0.81]	0.522
Fever	No	Yes	0.24 [−0.46–0.93]	0.504
Cough	No	Yes	0.58 [−0.10–1.26]	0.097
Dyspnea	No	Yes	0.50 [−0.53–1.53]	0.341
Headache	No	Yes	1.04 [0.37–1.71]	0.003
Myalgia	No	Yes	0.19 [−0.50–0.88]	0.589
Grasto-intestinal symptoms	No	Yes	−0.98 [−2.17–0.22]	0.112
Anosmia	No	Yes	−0.29 [−0.99–0.41]	0.423
Ageusia	No	Yes	−0.25 [−0.95–0.46]	0.497

*CV: continuous variable.*

Concerning the time between positive RT-PCR and symptom onset, a non-significant decrease of −0.12 log_10_ copies/mL [IQR −0.34–0.10] was observed. First, an increase 0.26 log_10_ copies/mL [IQR −0.67–1.18] was observed at 3–5 days compared with 0–2 days. Then, a decrease of −0.39 log_10_ copies/mL [IQR −1.70–0.92] was observed at 6–8 days.

Between no-symptoms patients with an initially salivary SARS-CoV-2 load of 4.01 log_10_ copies/mL [IQR 0.56–5.75] and patients with symptoms (Table 3), there was no significant median difference in the viral load (Table 4). However, COVID-19 adults declaring 5–6 or 7–9 symptoms have a higher viral median difference charge of 0.56 log_10_ copies/mL [IQR −0.71–1.83] and 0.50 log_10_ copies/mL [IQR −0.85–1.85] respectively.

The salivary SARS-CoV-2 viral load was consistently similar for the majority of the clinical symptoms concerned even if SARS-CoV-2 salivary load increased in presence of fever 0.24 log_10_ copies/mL [IQR −0.46–0.93], cough 0.58 log_10_ copies/mL [IQR −0.10–1.26], dyspnea 0.50 log_10_ copies/mL [IQR −0.53–1.53] and myalgia 0.19 log_10_ copies/mL [IQR −0.50–0.88]. With a median difference increase of 1.04 log_10_ copies/mL [IQR 0.37–1.71], headache in confirmation of symptomatic cases influence significantly the salivary viral load (*P* < 0.003) (Table 4). The patients without headache had a mean salivary viral load of 3.78 log_10_ copies/mL [IQR 1.95–4.58] whereas patients with headache had a mean salivary viral load of 4.51 log_10_ copies/mL [IQR 3.32–5.59] (Table 3).

In multivariate logistic regression analysis, the relation between salivary viral load and headache and GI symptoms remained significant after adjustment for age and sex (*P* = 0.004 and *P* = 0.043, respectively) (Table 5).

**TABLE 5:** Association of viral load with sex, age, symptoms of severe acute respiratory syndrome coronavirus 2 (SARS-CoV-2) infection: Multivariable Analysis.

Variable	References level	Class level	Mean difference [95% CI]	*p*-value
**Age**	CV		0.01 [−0.01–0.04]	0.188
**Gender**	Male	Female	−0.56 [−1.23–0.11]	0.102
**Symptoms**				
Cough	No	Yes	0.40 [−0.28–1.08]	0.250
Headache	No	Yes	1.02 [0.34–1.7]	0.004
Grasto-intestinal symptoms	No	Yes	−1.22 [−2.39—0.05]	0.043

*CV, continuous variable.*

## Discussion

The present study was designed to explore the quantification of SARS-CoV-2 in pure saliva of adults with asymptomatic to mild COVID-19. This robust multicenter observational trial, including 160 individuals, is the first, to our knowledge, to target this specific population—mean age of patients was 43.62 ± 15.56 years- and to describe the sociodemographic and clinical characteristics of the salivary viral load. The load SARS-CoV-2 value of the whole sample ranged from 0.00 to 10.19 with a median of 4.12 log_10_ copies/mL (IQR 2.95–5.16 log_10_ copies/mL), 27/159 patients (16.98%) had no charge. Between no-symptoms patients with an initially salivary SARS and patients with symptoms, there was no significant median difference in the viral load. Collectively, these data show that the oral cavity is an important site for SARS-CoV-2 infection and implicate saliva as a potential route of SARS-CoV-2 transmission.

While self-collected saliva is a realistic alternative option for the diagnosis of COVID-19, there are no significant studies in the literature evaluating the salivary viral load of adults with asymptomatic, to mild COVID-19. If Huang et al. (2021) suggest two possible sources for SARS-CoV-2 in saliva: an acellular fraction from infected glands making virus *de novo* and a cellular fraction from infected and shed oral mucosa, little is known about the correlation between viral load and age, gender, symptoms, and comorbidity. The first study quantifying the salivary load of SARS-CoV-2 included 12 hospitalized patients with laboratory-confirmed COVID-19, with a median age of 62.5 years. SARS-CoV-2 was detected in 91.7% of throat saliva samples from COVID-19 patients, and the number was as high as 1–2 × 10^8^ infectious copies per milliliter (To et al., 2020). Observational studies are subject to a number of different biases (Accorsi et al., 2021). Lecture of meta-analysis of diagnostic results from saliva (Moreira et al., 2021) reveals that no studies met our objective and methodological criteria.

So, studies included different subgroups categorized by gradation of disease severity, primarily in patients who developed severe disease admitted to intensive care during their hospital stay, and both inpatients with moderate disease symptoms (Alteri et al., 2020; Jeong et al., 2020; Kam et al., 2020; Kim et al., 2020; Lai et al., 2020; Pan et al., 2020; Pujadas et al., 2020; To et al., 2020; Yoon et al., 2020; Yu et al., 2020; Abasiyanik et al., 2021; Chua et al., 2021; Han et al., 2021; Hasanoglu et al., 2021). Then, participants were included with a diverse range of COVID-19 disease severity, including in majority cases hospitalized, and individuals with resolved infection. So, different diagnosis methods were used in self-collected saliva as nucleic acid amplification testing, transcription mediated amplification, reverse transcription loop-mediated isothermal amplification, TRIzol-based RNA extraction, despite quantitative reverse transcription PCR is the diagnostic standard for SARS-CoV-2 (Nagura-Ikeda et al., 2020). Several papers used the naive Ct values from qualitative RT-PCR as a quantitation unit or use the ΔCt values with incorrect quantitation unit (Shen et al., 2020; Zou et al., 2020). Saliva collection protocols for included studies were assessed for differences with respect to asking patients to collect pure saliva or to cough or clear their throat before submission of enhanced sample likely mixed sputum and saliva specimen or deep throat saliva specimen or requesting the patients submit “drool” or “spit” (Carrouel et al., 2020; Khiabani and Amirzade-Iranaq, 2021).

With reference to viral load during infection in asymptomatic or mildly affected adults with COVID-19, we observed that viral load is generally high, although asymptomatic viral loads are not statistically different from symptomatic viral loads, tending, however, to be lower. These results highlight the potential infectivity of saliva although the possible threshold of transmissibility is not specified and there is no standard reference in this regard. In particular, these results suggest that expelled oral droplets containing infectious virus and infected cells may be a source of airborne transmission of SARS-CoV-2. SARS-CoV-2 transmission from people who are either asymptomatic or mild has implications for prevention. Social distancing measures will need to be sustained at some level because droplet transmission from close contact with people with asymptomatic and mild infection occurs (Bazant and Bush, 2021). Easing of restrictions will, however, only be possible with wide access to testing, contact tracing, and rapid isolation of infected individuals.

The mean age of patients in our study was 43.6 years. We found that gender and age are not factors affecting viral load. According to published data, younger patients would be more likely to be asymptomatic than older patients (Li Y. et al., 2020). Studies have shown that both older age and male gender are associated with severe disease (Qian et al., 2020). To et al. (2020) found similar results to Zheng et al. (2020) and both concluded that older age is associated with higher viral load. Mahallawi et al. (2021) studied the viral load of lower and upper respiratory tract specimens from 3,006 COVID-19 positive patients. They found no statistically significant difference between age groups, while the viral load was statistically significantly higher in women than in men.

Specific observational studies as well as modeling work have shown that the infection can be asymptomatic or paucisymptomatic (causing little or no clinical manifestations) in 30–60% of infected individuals, especially in young adults and adults (Plucinski et al., 2021). Asymptomatic individuals were the source for 69% (20–85%) of all infections (Emery et al., 2020). Saliva from asymptomatic individuals contains infectious virus. Surprisingly, the viral load was found to be significantly similar between asymptomatic and symptomatic patients infected with SARS-CoV-2 (*p* = 0.573). Since the beginning of the pandemic, there has been controversy about the infectivity of asymptomatic patients. With the aforementioned limitation on the robustness of existing studies which are based on considerably smaller data sets, most studies demonstrate more rapid viral clearance in asymptomatic individuals than in symptomatic ones (McEvoy et al., 2021). A large representative sample with longitudinal data has shown that both symptomatic and asymptomatic patients are often characterized by a similar amount of virus at the onset of infection (Lavezzo et al., 2020). It is reported that approximately 40–45% of patients infected with SARS-CoV-2 will remain asymptomatic (Oran and Topol, 2020). Additionally, individuals with mild, non-specific, asymptomatic symptoms are difficult to identify and quarantine (Han et al., 2021). The ambiguity surrounding both asymptomatic and presymptomatic conditions is highlighted. They are described as suggestive, and both inconclusive. Because of the high risk of silent spread by asymptomatic individuals, it is imperative that screening programs include those without symptoms (Oran and Topol, 2020; Almadhi et al., 2021).

Screening from symptoms could prioritize tests and increase diagnostic sensitivity (Lan et al., 2020). However, in our study, salivary load quantification is not discriminated on the number and nature of reported symptoms except for headache. General non-respiratory symptoms (eye pain, muscle pain, general malaise, fever, headache, and extreme fatigue), although not very specific, are the strongest independent predictors of positive tests (Tostmann et al., 2020). As a non-specific symptom, headache can occur not only in COVID-19 but also in other viral diseases. Therefore, headache alone may not raise suspicion of SARS-CoV-2 infection although headache was 1.7 times more common in patients with COVID-19 respiratory viral infection than in those with non-COVID-19 respiratory viral infection with *p* = 0.04 (Correia et al., 2020; Mutiawati et al., 2021). The presence of gastrointestinal symptoms associated with salivary viral load in COVID-19 patients raises questions about the impact of COVID-19 infection on the quality of life of at-risk subjects. It is quite plausible to observe the development of several gastro-intestinal symptoms induced by SARS-CoV-2 infection in patients, ranging from nausea, vomiting, and diarrhea to loss of appetite and abdominal pain (Yusuf et al., 2021).

Chemosensory dysfunctions, especially hyposmia, anosmia, hypogeusia, and ageusia, are one of the major symptoms of SARS-CoV2 infection (Srinivasan, 2021). Self-reported loss of smell and taste is a better prognosticator than other symptoms such as cough, fatigue, or fever for predicting symptomatic infection (Mastrangelo et al., 2021). Our results indicate that SARS-CoV-2 levels in saliva do not correlate with taste alterations.

First limitation of our study is that it focused on the second wave of the epidemic in the second half of 2020. Adaptation of salivary quantification guidelines in relation to newly detected SARS-CoV-2 variants is necessary. At the time of the study the variant circulating in France was predominantly the UK variant (B.1.1.7) and not the Delta variant (B.1.617.2) which is now detected in the majority of cases. Thus, it is likely that the results would be different if the study were conducted now since the delta variant has different characteristics (Mlcochova et al., 2021).

Secondly, we also clearly explained the ambiguity surrounding asymptomatic vs. presymptomatic status. We describe them as suggestive, not conclusive. While measuring viral load can be useful in clinical practice, a positive RT-qPCR result does not necessarily mean that the person is still infectious or still has significant disease. The RNA could be from a non-viable virus and/or the amount of live virus could be too low to allow transmission (Trunfio et al., 2021). Contacts of a carrier with a high viral salivary load may have a higher risk of acquiring infection, but to date there is no evidence that acquired infection will be more likely to be symptomatic or severe (Li R. et al., 2020; Hasanoglu et al., 2021; van Kampen et al., 2021).

Strengths of our study is that, although *post hoc* studies have revealed the importance of SARS-CoV-2 corona virus 2 transmission from both asymptomatic and mildly symptomatic cases, the virologic basis for their infectivity remains largely unquantified (Jones et al., 2021). Despite PCR method do not measure infectious virus, our study produces original and robust quantitative data applied to an ambulatory adult population susceptible to be the prime actor of transmission until effective vaccines have been distributed widely.

## Conclusion

Our results raise the possibility that the oral cavity is an important site for SARS-CoV-2 infection and implicate saliva as a potential route of SARS-CoV-2 transmission. This result may have public health implications if enhanced saliva samples are used for asymptomatic screening. Considering oral SARS-CoV-2 infection and the ease of saliva for transmission, it remains critical to further understanding of the dominant modes of viral spread across the spectrum of asymptomatic, pre-symptomatic and symptomatic individuals.

## Data Availability Statement

The raw data supporting the conclusions of this article will be made available by the authors, without undue reservation.

## Ethics Statement

The studies involving human participants were reviewed and approved by the Committee for the Protection of Persons South Mediterranean III, France. The patients/participants provided their written informed consent to participate in this study.

## Author Contributions

FC, DB, and CD proposed the original study idea and designed the trial and study protocol. DB and FC contributed to the data interpretation and wrote the first draft of the manuscript. FC, CD, and HP verified the data. EG, AE, ML, and JD were responsible for the site work including the recruitment, follow up and data collection. HP monitored the trial. DB did the main analysis. CD, EG, and JD contributed to the revision of the manuscript. All authors reviewed and accepted the manuscript before submission.

## Conflict of Interest

The authors declare that the research was conducted in the absence of any commercial or financial relationships that could be construed as a potential conflict of interest.

## Publisher’s Note

All claims expressed in this article are solely those of the authors and do not necessarily represent those of their affiliated organizations, or those of the publisher, the editors and the reviewers. Any product that may be evaluated in this article, or claim that may be made by its manufacturer, is not guaranteed or endorsed by the publisher.

## References

[B1] Abasiyanik M. F. Flood B. Lin J. Ozcan S. Rouhani S. J. Pyzer A. (2021). Sensitive detection and quantification of SARS-CoV-2 in saliva. *Sci. Rep.* 11:12425. 10.1038/s41598-021-91835-7 34127708 PMC8203799

[B2] Accorsi E. K. Qiu X. Rumpler E. Kennedy-Shaffer L. Kahn R. Joshi K. (2021). How to detect and reduce potential sources of biases in studies of SARS-CoV-2 and COVID-19. *Eur. J. Epidemiol.* 36 179–196. 10.1007/s10654-021-00727-7 33634345 PMC7906244

[B3] Almadhi M. A. Abdulrahman A. Sharaf S. A. AlSaad D. Stevenson N. J. Atkin S. L. (2021). The high prevalence of asymptomatic SARS-CoV-2 infection reveals the silent spread of COVID-19. *Int. J. Infect. Dis.* 105 656–661. 10.1016/j.ijid.2021.02.100 33647516 PMC7908846

[B4] Alteri C. Cento V. Antonello M. Colagrossi L. Merli M. Ughi N. (2020). Detection and quantification of SARS-CoV-2 by droplet digital PCR in real-time PCR negative nasopharyngeal swabs from suspected COVID-19 patients. *PLoS One* 15:e0236311. 10.1371/journal.pone.0236311 32898153 PMC7478621

[B5] Bazant M. Z. Bush J. W. M. (2021). A guideline to limit indoor airborne transmission of COVID-19. *Proc. Natl. Acad. Sci. U.S.A.* 118:e2018995118. 10.1073/pnas.2018995118 33858987 PMC8092463

[B6] Caixeta D. C. Oliveira S. W. Cardoso-Sousa L. Cunha T. M. Goulart L. R. Martins M. M. (2021). One-year update on salivary diagnostic of COVID-19. *Front. Public Health* 9:589564. 10.3389/fpubh.2021.589564 34150692 PMC8210583

[B7] Carrouel F. Valette M. Perrier H. Bouscambert-Duchamp M. Dussart C. Tramini P. (2021). Performance of self-collected saliva testing compared with nasopharyngeal swab testing for the detection of SARS-CoV-2. *Viruses* 13:895. 10.3390/v13050895 34066046 PMC8150338

[B8] Carrouel F. Viennot S. Valette M. Cohen J.-M. Dussart C. Bourgeois D. (2020). Salivary and Nasal detection of the SARS-CoV-2 virus after antiviral mouthrinses (BBCovid): a structured summary of a study protocol for a randomised controlled trial. *Trials* 21:906. 10.1186/s13063-020-04846-6 33138848 PMC7604647

[B9] Ceron J. J. Lamy E. Martinez-Subiela S. Lopez-Jornet P. Capela-Silva F. Eckersall P. D. (2020). Use of saliva for diagnosis and monitoring the SARS-CoV-2: a general perspective. *J. Clin. Med.* 9:1491. 10.3390/jcm9051491 32429101 PMC7290439

[B10] Chua G. T. Wong J. S. C. To K. K. W. Lam I. C. S. Yau F. Y. S. Chan W. H. (2021). Saliva viral load better correlates with clinical and immunological profiles in children with coronavirus disease 2019. *Emerg Microbes Infect.* 10 235–241. 10.1080/22221751.2021.1878937 33467982 PMC7899683

[B11] Correia A. O. Feitosa P. W. G. Moreira J. L. de S. Nogueira S. ÁR. Fonseca R. B. (2020). Neurological manifestations of COVID-19 and other coronaviruses: a systematic review. *Neurol. Psychiatry Brain Res.* 37 27–32. 10.1016/j.npbr.2020.05.008 32834527 PMC7261450

[B12] Emery J. C. Russell T. W. Liu Y. Hellewell J. Pearson C. A. Cmmid Covid-19 Working Group (2020). The contribution of asymptomatic SARS-CoV-2 infections to transmission on the diamond princess cruise ship. *Elife* 9:e58699. 10.7554/eLife.58699 32831176 PMC7527238

[B13] Han M. S. Byun J.-H. Cho Y. Rim J. H. (2021). RT-PCR for SARS-CoV-2: quantitative versus qualitative. *Lancet Infect. Dis.* 21:165. 10.1016/S1473-3099(20)30424-2 PMC7239624 32445709

[B14] Hart W. S. Maini P. K. Thompson R. N. (2021). High infectiousness immediately before COVID-19 symptom onset highlights the importance of continued contact tracing. *Elife* 10:e65534. 10.7554/eLife.65534 33899740 PMC8195606

[B15] Hasanoglu I. Korukluoglu G. Asilturk D. Cosgun Y. Kalem A. K. Altas A. B. (2021). Higher viral loads in asymptomatic COVID-19 patients might be the invisible part of the iceberg. *Infection* 49 117–126. 10.1007/s15010-020-01548-8 33231841 PMC7685188

[B16] Huang N. Pérez P. Kato T. Mikami Y. Okuda K. Gilmore R. C. (2021). SARS-CoV-2 infection of the oral cavity and saliva. *Nat. Med.* 27 892–903. 10.1038/s41591-021-01296-8 33767405 PMC8240394

[B17] Jacot D. Greub G. Jaton K. Opota O. (2020). Viral load of SARS-CoV-2 across patients and compared to other respiratory viruses. *Microbes Infect.* 22 617–621. 10.1016/j.micinf.2020.08.004 32911086 PMC7476607

[B18] Jeong H. W. Kim S.-M. Kim H.-S. Kim Y.-I. Kim J. H. Cho J. Y. (2020). Viable SARS-CoV-2 in various specimens from COVID-19 patients. *Clin. Microbiol. Infect.* 26 1520–1524. 10.1016/j.cmi.2020.07.020 32711057 PMC7375961

[B19] Jones T. C. Biele G. Mühlemann B. Veith T. Schneider J. Beheim-Schwarzbach J. (2021). Estimating infectiousness throughout SARS-CoV-2 infection course. *Science* 373:eabi5273. 10.1126/science.abi5273 34035154 PMC9267347

[B20] Kam K.-Q. Yung C. F. Maiwald M. Chong C. Y. Soong H. Y. Loo L. H. (2020). Clinical utility of buccal swabs for severe acute respiratory syndrome coronavirus 2 detection in coronavirus disease 2019-infected children. *J. Pediatr. Infect. Dis. Soc.* 9 370–372. 10.1093/jpids/piaa068 32463086 PMC7313923

[B21] Khiabani K. Amirzade-Iranaq M. H. (2021). Are saliva and deep throat sputum as reliable as common respiratory specimens for SARS-CoV-2 detection? A systematic review and meta-analysis. *Am. J. Infect. Control* 49 1165–1176. 10.1016/j.ajic.2021.03.008 33774101 PMC7987587

[B22] Kim S. E. Lee J. Y. Lee A. Kim S. Park K. H. Jung S. I. (2020). Viral load kinetics of SARS-CoV-2 infection in saliva in korean patients: a prospective multi-center comparative study. *J. Korean Med. Sci.* 35:e287. 10.3346/jkms.2020.35.e287 32776725 PMC7415999

[B23] Lai C. K. C. Chen Z. Lui G. Ling L. Li T. Wong M. C. S. (2020). Prospective study comparing deep throat saliva with other respiratory tract specimens in the diagnosis of novel coronavirus disease 2019. *J. Infect. Dis.* 222 1612–1619. 10.1093/infdis/jiaa487 32738137 PMC7454747

[B24] Lan F.-Y. Filler R. Mathew S. Buley J. Iliaki E. Bruno-Murtha L. A. (2020). COVID-19 symptoms predictive of healthcare workers’ SARS-CoV-2 PCR results. *PLoS One* 15:e0235460. 10.1371/journal.pone.0235460 32589687 PMC7319316

[B25] Lavezzo E. Franchin E. Ciavarella C. Cuomo-Dannenburg G. Barzon L. Del Vecchio C. (2020). Suppression of a SARS-CoV-2 outbreak in the Italian municipality of Vo’. *Nature* 584 425–429. 10.1038/s41586-020-2488-1 32604404 PMC7618354

[B26] Li R. Pei S. Chen B. Song Y. Zhang T. Yang W. (2020). Substantial undocumented infection facilitates the rapid dissemination of novel coronavirus (SARS-CoV-2). *Science* 368 489–493. 10.1126/science.abb3221 32179701 PMC7164387

[B27] Li Y. Shi J. Xia J. Duan J. Chen L. Yu X. (2020). Asymptomatic and symptomatic patients with non-severe coronavirus disease (COVID-19) have similar clinical features and virological courses: a retrospective single center study. *Front. Microbiol.* 11:1570. 10.3389/fmicb.2020.01570 32754137 PMC7344298

[B28] Mahallawi W. H. Alsamiri A. D. Dabbour A. F. Alsaeedi H. Al-Zalabani A. H. (2021). Association of Viral Load in SARS-CoV-2 patients with age and gender. *Front. Med.* 8:608215. 10.3389/fmed.2021.608215 33585523 PMC7873591

[B29] Mastrangelo A. Bonato M. Cinque P. (2021). Smell and taste disorders in COVID-19: from pathogenesis to clinical features and outcomes. *Neurosci. Lett.* 748:135694. 10.1016/j.neulet.2021.135694 33600902 PMC7883672

[B30] McEvoy D. McAloon C. Collins A. Hunt K. Butler F. Byrne A. (2021). Relative infectiousness of asymptomatic SARS-CoV-2 infected persons compared with symptomatic individuals: a rapid scoping review. *BMJ Open* 11:e042354. 10.1136/bmjopen-2020-042354 33947725 PMC8098293

[B31] Mlcochova P. Kemp S. A. Dhar M. S. Papa G. Meng B. Ferreira I. A. T. M. (2021). SARS-CoV-2 B.1.617.2 delta variant replication and immune evasion. *Nature* 599 114–119. 10.1038/s41586-021-03944-y 34488225 PMC8566220

[B32] Moreira V. M. Mascarenhas P. Machado V. Botelho J. Mendes J. J. Taveira N. (2021). Diagnosis of SARS-Cov-2 infection by RT-PCR using specimens other than naso- and oropharyngeal swabs: a systematic review and meta-analysis. *Diagnostics* 11:363. 10.3390/diagnostics11020363 33670020 PMC7926389

[B33] Mutiawati E. Syahrul S. Fahriani M. Fajar J. K. Mamada S. S. Maliga H. A. (2021). Global prevalence and pathogenesis of headache in COVID-19: a systematic review and meta-analysis. *F1000Res* 9:1316. 10.12688/f1000research.27334.2 33953911 PMC8063523

[B34] Nagura-Ikeda M. Imai K. Tabata S. Miyoshi K. Murahara N. Mizuno T. (2020). Clinical evaluation of self-collected saliva by quantitative reverse transcription-PCR (RT-qPCR), Direct RT-qPCR, reverse transcription-loop-mediated isothermal amplification, and a rapid antigen test to diagnose COVID-19. *J. Clin. Microbiol.* 58:e01438-20. 10.1128/JCM.01438-20 32636214 PMC7448663

[B35] Nasserie T. Hittle M. Goodman S. N. (2021). Assessment of the frequency and variety of persistent symptoms among patients with COVID-19: a systematic review. *JAMA Netw. Open* 4:e2111417. 10.1001/jamanetworkopen.2021.11417 34037731 PMC8155823

[B36] Oran D. P. Topol E. J. (2020). Prevalence of asymptomatic SARS-CoV-2 infection. *Ann. Intern. Med.* M20-3012. 10.7326/M20-3012 33587872

[B37] Pan Y. Zhang D. Yang P. Poon L. L. M. Wang Q. (2020). Viral load of SARS-CoV-2 in clinical samples. *Lancet Infect. Dis.* 20 411–412. 10.1016/S1473-3099(20)30113-4 32105638 PMC7128099

[B38] Plucinski M. M. Wallace M. Uehara A. Kurbatova E. V. Tobolowsky F. A. Schneider Z. D. (2021). Coronavirus disease 2019 (COVID-19) in Americans aboard the diamond princess cruise ship. *Clin. Infect. Dis.* 72 e448–e457. 10.1093/cid/ciaa1180 32785683 PMC7454359

[B39] Pujadas E. Chaudhry F. McBride R. Richter F. Zhao S. Wajnberg A. (2020). SARS-CoV-2 viral load predicts COVID-19 mortality. *Lancet Respir. Med.* 8:e70. 10.1016/S2213-2600(20)30354-4 PMC7836878 32771081

[B40] Qian J. Zhao L. Ye R.-Z. Li X.-J. Liu Y.-L. (2020). Age-dependent gender differences in COVID-19 in Mainland China: comparative study. *Clin. Infect. Dis.* 71 2488–2494. 10.1093/cid/ciaa683 32473009 PMC7314154

[B41] Sapkota D. Søland T. M. Galtung H. K. Sand L. P. Giannecchini S. To K. K. W. (2020). COVID-19 salivary signature: diagnostic and research opportunities. *J. Clin. Pathol.* jclinpath-2020-206834. 10.1136/jclinpath-2020-206834 32769214

[B42] Shen C. Wang Z. Zhao F. Yang Y. Li J. Yuan J. (2020). Treatment of 5 critically Ill patients with COVID-19 with convalescent plasma. *JAMA* 323 1582–1589. 10.1001/jama.2020.4783 32219428 PMC7101507

[B43] Srinivasan M. (2021). Taste dysfunction and long COVID-19. *Front. Cell Infect. Microbiol* 11:716563. 10.3389/fcimb.2021.716563 34336725 PMC8317431

[B44] To K. K.-W. Tsang O. T.-Y. Leung W.-S. Tam A. R. Wu T.-C. Lung D. C. (2020). Temporal profiles of viral load in posterior oropharyngeal saliva samples and serum antibody responses during infection by SARS-CoV-2: an observational cohort study. *Lancet Infect. Dis.* 20 565–574. 10.1016/S1473-3099(20)30196-1 32213337 PMC7158907

[B45] Tostmann A. Bradley J. Bousema T. Yiek W.-K. Holwerda M. Bleeker-Rovers C. (2020). Strong associations and moderate predictive value of early symptoms for SARS-CoV-2 test positivity among healthcare workers, the Netherlands, March 2020. *Euro Surveill* 25:2000508. 10.2807/1560-7917.ES.2020.25.16.2000508 32347200 PMC7189649

[B46] Trunfio M. Longo B. M. Alladio F. Venuti F. Cerutti F. Ghisetti V. (2021). On the SARS-CoV-2 “variolation hypothesis”: no association between viral load of index cases and COVID-19 severity of secondary cases. *Front. Microbiol.* 12:646679. 10.3389/fmicb.2021.646679 33815334 PMC8010676

[B47] van Kampen J. J. A. van de Vijver D. A. M. C. Fraaij P. L. A. Haagmans B. L. Lamers M. M. Okba N. (2021). Duration and key determinants of infectious virus shedding in hospitalized patients with coronavirus disease-2019 (COVID-19). *Nat. Commun.* 12:267. 10.1038/s41467-020-20568-4 33431879 PMC7801729

[B48] Vasudevan H. N. Xu P. Servellita V. Miller S. Liu L. Gopez A. (2021). Digital droplet PCR accurately quantifies SARS-CoV-2 viral load from crude lysate without nucleic acid purification. *Sci. Rep.* 11:780. 10.1038/s41598-020-80715-1 33436939 PMC7804156

[B49] Walsh K. A. Jordan K. Clyne B. Rohde D. Drummond L. Byrne P. (2020). SARS-CoV-2 detection, viral load and infectivity over the course of an infection. *J. Infect.* 81 357–371. 10.1016/j.jinf.2020.06.067 32615199 PMC7323671

[B50] Yoon J. G. Yoon J. Song J. Y. Yoon S. Y. Lim C. S. Seong H. (2020). Clinical significance of a high SARS-CoV-2 viral load in the saliva. *J. Korean Med. Sci.* 35:e195. 10.3346/jkms.2020.35.e195 32449329 PMC7246183

[B51] Yu F. Yan L. Wang N. Yang S. Wang L. Tang Y. (2020). Quantitative detection and viral load analysis of SARS-CoV-2 in infected patients. *Clin. Infect. Dis.* 71 793–798. 10.1093/cid/ciaa345 32221523 PMC7184442

[B52] Yusuf F. Fahriani M. Mamada S. S. Frediansyah A. Abubakar A. Maghfirah D. (2021). Global prevalence of prolonged gastrointestinal symptoms in COVID-19 survivors and potential pathogenesis: a systematic review and meta-analysis. *F1000Res* 10:301. 10.12688/f1000research.52216.1 34131481 PMC8171196

[B53] Zheng S. Fan J. Yu F. Feng B. Lou B. Zou Q. (2020). Viral load dynamics and disease severity in patients infected with SARS-CoV-2 in Zhejiang province, China, January-March 2020: retrospective cohort study. *BMJ* 369:m1443. 10.1136/bmj.m1443 32317267 PMC7190077

[B54] Zou L. Ruan F. Huang M. Liang L. Huang H. Hong Z. (2020). SARS-CoV-2 viral load in upper respiratory specimens of infected patients. *N. Engl. J. Med.* 382 1177–1179. 10.1056/NEJMc2001737 32074444 PMC7121626

